# Kinetic Fingerprinting Links Bacteria-Phage Interactions with Emergent Dynamics: Rapid Depletion of *Klebsiella pneumoniae* Indicates Phage Synergy

**DOI:** 10.3390/antibiotics9070408

**Published:** 2020-07-14

**Authors:** Holger Loessner, Insea Schlattmeier, Marie Anders-Maurer, Isabelle Bekeredjian-Ding, Christine Rohde, Johannes Wittmann, Cornelia Pokalyuk, Oleg Krut, Christel Kamp

**Affiliations:** 1 Paul-Ehrlich-Institut, 63225 Langen, Germany; holger.loessner@pei.de (H.L.); marie.anders-maurer@pei.de (M.A.-M.); isabelle.bekeredjian-ding@pei.de (I.B.-D.); oleg.krut@pei.de (O.K.); 2 Goethe University Frankfurt, Institute of Mathematics, 60325 Frankfurt, Germany; schlattmeier@math.uni-frankfurt.de (I.S.); pokalyuk@math.uni-frankfurt.de (C.P.); 3 Leibniz Institute DSMZ German Collection of Microorganisms and Cell Cultures, 38124 Braunschweig, Germany; chr@dsmz.de (C.R.); jow12@dsmz.de (J.W.)

**Keywords:** population dynamics, mathematical modeling, *Klebsiella pneumoniae*, phage, emergence, resistance, synergistic interaction, kinetic fingerprint, phage therapy

## Abstract

The specific temporal evolution of bacterial and phage population sizes, in particular bacterial depletion and the emergence of a resistant bacterial population, can be seen as a *kinetic fingerprint* that depends on the manifold interactions of the specific phage–host pair during the course of infection. We have elaborated such a kinetic fingerprint for a human urinary tract *Klebsiella pneumoniae* isolate and its phage vB_KpnP_Lessing by a modeling approach based on data from *in vitro* co-culture. We found a faster depletion of the initially sensitive bacterial population than expected from simple mass action kinetics. A possible explanation for the rapid decline of the bacterial population is a synergistic interaction of phages which can be a favorable feature for phage therapies. In addition to this interaction characteristic, analysis of the kinetic fingerprint of this bacteria and phage combination revealed several relevant aspects of their population dynamics: A reduction of the bacterial concentration can be achieved only at high multiplicity of infection whereas bacterial extinction is hardly accomplished. Furthermore the binding affinity of the phage to bacteria is identified as one of the most crucial parameters for the reduction of the bacterial population size. Thus, kinetic fingerprinting can be used to infer phage–host interactions and to explore emergent dynamics which facilitates a rational design of phage therapies.

## 1. Introduction

A century after their discovery, phages—viruses that infect bacteria—have regained attention as highly specific anti-microbial agents for the treatment of multidrug-resistant (MDR) bacterial pathogens where common broad-spectrum antibiotic drugs increasingly fail [1,2,3,4]. MDR strains of highest clinical concern have been referred to as “ESKAPE” pathogens, encompassing strains of *Enterococcus faecium, Staphylococcus aureus, Klebsiella pneumoniae, Acinetobacter baumannii, Pseudomonas aeruginosa*, and *Enterobacter* spp. [5,6]. Clinical interventions using therapeutic phages have been performed for a long time based on experience and empiricism with successes in many different bacterial infections [7], but also ambiguous results or failure of phage therapy have frequently been observed [8]. In order to advance a rational, safe, and efficacious application of phage therapy the characteristics of specific phage–pathogen interactions must be unraveled in order to predict and tailor conditions for the application of therapeutic phages to individual patients. For example, phage doses applied to the patient and their repeated administration have to be specified [9]. Eventually, safety and efficacy of treatment regimens must be proven in clinical trials before phage therapy medicinal products become available for routine use in medical practice [10].

Phages are ubiquitously present in nature and can display complex life styles and manifold interactions with susceptible bacteria depending on environmental conditions which are a matter of constant change and evolution [11,12]. In phage therapy mostly phages are used which are strictly lytic and lack virulence, resistance, or toxic determinants [13]. The interaction between bacteria and lytic phage species is typically determined by fast emergence of bacterial resistance against phages which can undermine the success of phage therapy [14,15,16]. Thus, in phage therapy usually application of a high number of phages is anticipated that lies above the so called *inundation threshold*, which mediates a fast decline of target bacteria before resistant bacteria emerge and resume growth [14]. However, interaction dynamics of individual phage–host pairs vary depending on mechanisms of phage invasion, phage replication, burst size, and bacterial responses including bacterial immunity [11,17,18] and corresponding mechanisms of phage evasion [19]. In addition, environmental conditions such as available nutrients, microbiome/virome composition, and physico-chemical conditions impact infection courses [20]. Therefore, different inundation thresholds for specific phages, respective target bacteria and surrounding conditions are expected. As it is impossible to disentangle and control all interactions in a complex environment, we propose a bottom–up approach and study the interaction of one bacterial and corresponding phage species. The time scales for the emergence (and sustainment) of resistance strongly depend on the specific phage and bacterial host characteristics. The specific temporal evolution of bacterial and phage population sizes can be seen as a *kinetic fingerprint* which provides insights into the underlying dynamics including binding kinetics and mechanisms of bacterial resistance and phage evasion. The well-defined setting further allows for a prediction of the inundation threshold of the specific phage–host pair.

Here, we studied the *in vitro* infection course of a *K. pneumoniae* human urinary tract isolate (DSM 11678) and its phage vB_KpnP_Lessing. This phage displays a high sequence homology of about 95% to members of the *K. pneumonia* phage K32 family [21]. To decipher the kinetic fingerprint of the *K. pneumoniae* vB_KpnP_Lessing pair we built on modeling studies [22,23,24] which have implemented knowledge on phage–host interactions and thereby advanced the understanding of specific phage–bacteria interactions and ecological dynamics at large [17,18,25]. In particular, we focused on a model for a single bacterial strain and phage species [14] and extend it to allow for more flexibility in infection dynamics. Already, this simple system shows surprising dynamical features which indicate synergistic infection dynamics of vB_KpnP_Lessing phages in *K. pneumonia.* More precisely, the infection dynamics speeds up faster with growing phage concentrations than one would expect only from simple mass action kinetics, i.e., from assuming that the number of infected bacteria grows proportionally to the concentrations of bacteria and phages. The speed-up of infection dynamics indicates that the presence of one phage particle supports the infection dynamics of another phage particle (synergy), for which we discuss potential mechanisms.

## 2. Results

### 2.1. Modeling Bacteria Phage Interactions

In order to understand the dynamics of bacterial and phage populations, we further developed an approach based on [14] which allows us to understand the dynamics of bacterial and phage populations in terms of certain thresholds in bacterial and phage population sizes: The *proliferation threshold* $S_{P}$ defines a minimal bacterial concentration required to support phage proliferation, the *inundation threshold* $V_{I}$ represents the phage concentration required to deplete a population of sensitive bacteria.

**Figure 1:**
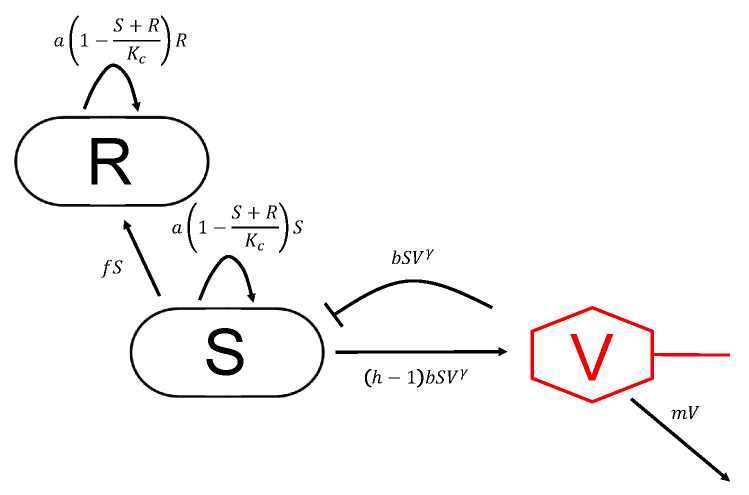
Interactions between bacteria and phages considered in the model: Phages infect sensitive bacteria (S) which are in consequence depleted by $bSV^{\gamma }$ resulting in a release of phages by $\left(h-1\right)bSV^{\gamma }$, i.e., with a burst size *h*. Sensitive bacteria become resistant to phages (*R*) at a rate *f*, all bacteria grow at a rate $a\left(1-\frac{S+R}{K_{c}}\right)$ that is restricted by the carrying capacity $K_{c}$. Phages decay at a rate *m*.

These thresholds as well as model parameters are estimated from experimental observations that we gained by adding phage vB_KpnP_Lessing in different multiplicities of infection (MOI) to an early log phase culture of *Klebsiella pneumoniae* strain DSM 11678. *Klebsiella pneumoniae* cultures showed a median concentration of $4.0\times 10^{8}$ colony forming units (cfu) per mL covering multiplicities of infection (MOI) from $10^{-4}$ to 1 in terms of plaque forming units (pfu) per mL. In order to describe our experimental data we further develop the model by Cairns et al. [14] as shown in Figure 1 considering the following interactions: Bacteria at concentration *S* can be infected by free phages at a concentration *V* with a binding parameter *b* and are subsequently depleted through lysis leading to a reduction by $bSV^{\gamma }$. Lysis goes along with the release of *h* new phage phage particles (burst size) increasing the population of free phages by $\left(h-1\right)bSV^{\gamma }$ (discounting for the phage starting the infection). Bacteria susceptible to infection (*S*) grow at a rate $a\left(1-\frac{S+R}{K_{c}}\right)$ which is limited by the carrying capacity $K_{c}$. Susceptible bacteria acquire resistance against phage infection at a rate *f* resulting in resistant bacteria (*R*). We assume identical growth rates for sensitive and resistant bacteria in the interest of parsimony. Analogously, estimates for a period of latency between the infection of bacteria and the release of new phages were at the time scale of minutes (data not shown). We consequently found that we could neglect latency on the time scale of the observed dynamics that reaches out over hours. Finally, free phages decay at a rate *m* and the whole model can be summarized by Equations (1)–(3) (cf. Materials and Methods).

The exponent γ in the model allows to describe the infection dynamics more flexibly than it has been possible by the earlier assumed mass action dynamics (e.g., in [14]). There, reaction rates depend linearly on the concentrations of components corresponding to γ=1. Other studies have observed values of γ<1, which have been associated with spatial heterogeneities in the system [17]. Values of γ>1 can be interpreted as synergistic infection dynamics in terms of an effective binding or more generally infection parameter that grows with phage concentration.

**Figure 2:**
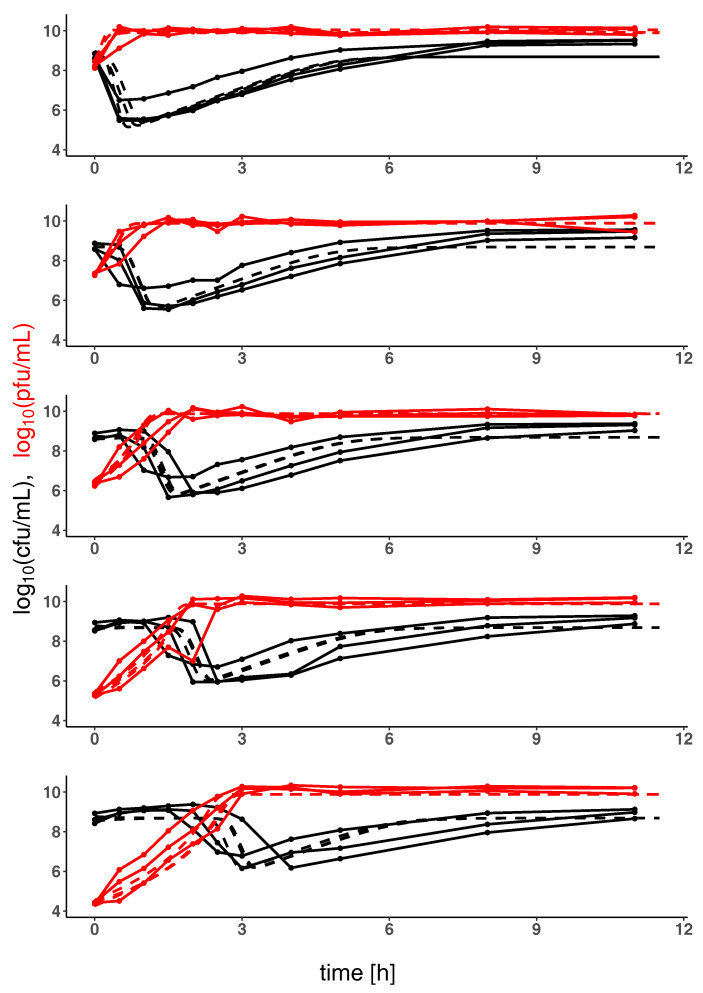
Kinetic profile showing population sizes of *K. pneumoniae* and of phage vB_KpnP_Lessing over time: The evolution of *K. pneumoniae* and the corresponding phage vB_KpnP_Lessing population sizes (in colony forming units (solid black lines, cfu mL${}^{-1}$) and plaque forming units (solid red lines, pfu mL${}^{-1}$)) over time (in hours) is shown in combination with the model prediction (for each experimental replicate, dashed lines). Kinetic profiles are shown for five different initial phage population sizes (decreasing from top to bottom) and for estimated optimal parameters listed in Table 1.

Parameters of the model shown in Figure 1 and defined through Equations (1)–(3) have been estimated based on experimental data of the temporal evolution of bacteria and phage population sizes as shown in Figure 2. Table 1 shows that optimal parameters are consistently estimated after initialization of the optimizer with random starting values, in particular indicating synergistic phage dynamics through γ>1. This means that with increasing phage concentration the bacterial lysis occurs more quickly than can be explained by simple mass action kinetics of the lytic infection cycle. Other factors seem to facilitate bacterial lysis. Notably these factors correlate with concentration of bacteriophage and therefore represent synergistic mechanisms of bacterial lysis.

**Figure 3:**
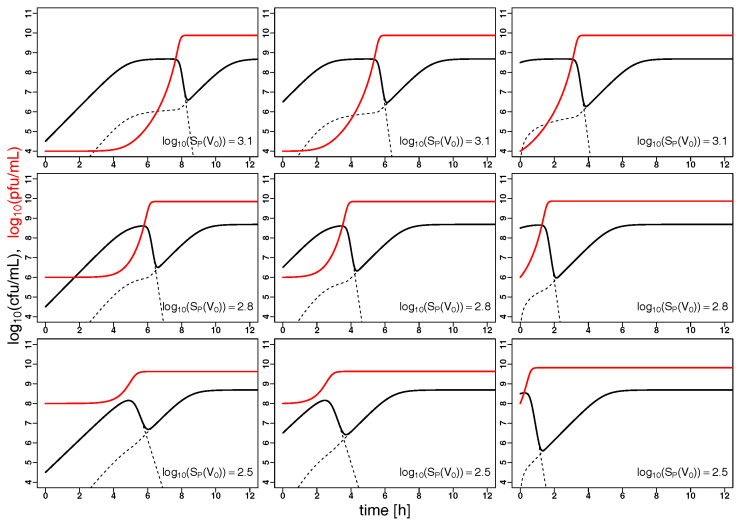
Phage synergy leads to fast bacterial depletion: The evolution of *K. pneumoniae* (solid black) and the corresponding phage vB_KpnP_Lessing (solid red) population sizes (in colony forming units (cfu mL${}^{-1}$) and plaque forming units (pfu mL${}^{-1}$)) over time (in hours) is extrapolated from the model based on the optimal parameters listed in Table 1 with varying starting concentrations, the right column corresponds to the setting in Figure 2. Dashed black lines correspond to the population sizes of sensitive and resistant bacteria. In all panels, the initial phage concentrations are below the inundation threshold $V_{I}$ (cf. Equation (4)), i.e., the minimal phage concentration required for a decline in sensitive bacteria. However, the initial concentrations of sensitive bacteria always exceed the proliferation threshold $S_{P}$ (cf. Equation (5)), i.e., the phage populations are growing.

Figure 2 shows *K. pneumoniae* and corresponding phage population sizes for varying initial multiplicity of infection (MOI) as well as model predictions with optimal parameters estimated from these data. The sharp initial decrease of bacterial population sizes at high initial phage concentrations reflects the synergistic phage dynamics represented by γ=1.15>1 in the model. Note that a minimal phage concentration $V_{I}$ is required for a depletion of sensitive bacteria (inundation threshold) which can be estimated for the system of *K. pneumoniae* and corresponding phage to be approximately $V_{I}^{max}={\left(\frac{a-f}{b}\right)}^{\frac{1}{\gamma }}=10^{9}$ pfu mL${}^{-1}$ (for $S+R\ll K_{c}$ and below otherwise, cf. Equation (4) and Figure 3). If the initial concentration of phages is below the inundation threshold phages may only slow bacterial growth but cannot induce their depletion. On the other hand, a phage population can only grow on a bacterial population if its concentration exceeds the proliferation threshold of $S_{P}\left(V\right)=\frac{m}{b\left(h-1\right)V^{\gamma -1}}$ which is passed in all our experiments ($S_{P}^{max}=\frac{m}{b(h-1)}=10^{3}$ cfu mL${}^{-1}$, cf. Equation (5)).

Note that our model does not allow for a reversion of phage resistance meaning that, according to the model, all bacteria surviving in the late stage of the experiment are phage resistant. Phages show a slow rate of natural decline *m* and in consequence persist over the observed time scale despite the dominance of resistant hosts.

### 2.2. Model Predictions

**Figure 4:**
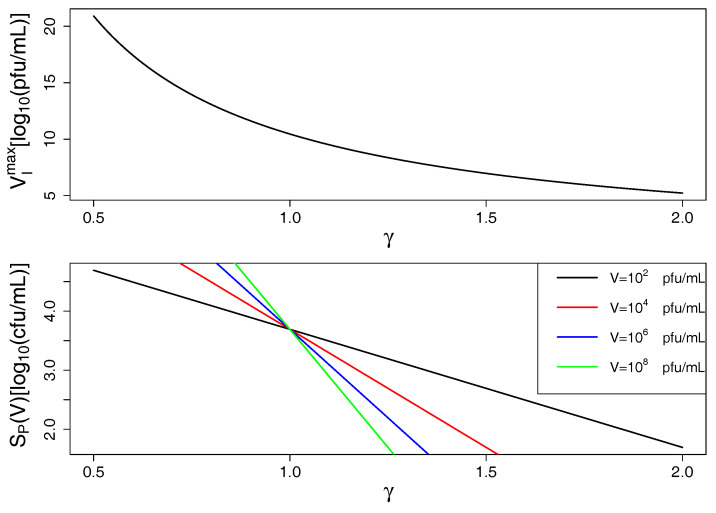
Inundation and proliferation thresholds decrease with growing γ (i.e., growing phage synergy): In the top panel, $V_{I}^{max}$ is plotted over γ (cf. Equation (4)) showing that smaller phage concentrations are sufficient to induce bacterial decline with growing phage synergy. In the bottom panel, $S_{P}$ is plotted over γ (cf. Equation (5)) showing that phages can proliferate on smaller bacterial populations with growing phage synergy (also depending on phage population size *V*). Model predictions are based on optimal parameters from Table 1.

**Figure A1:**
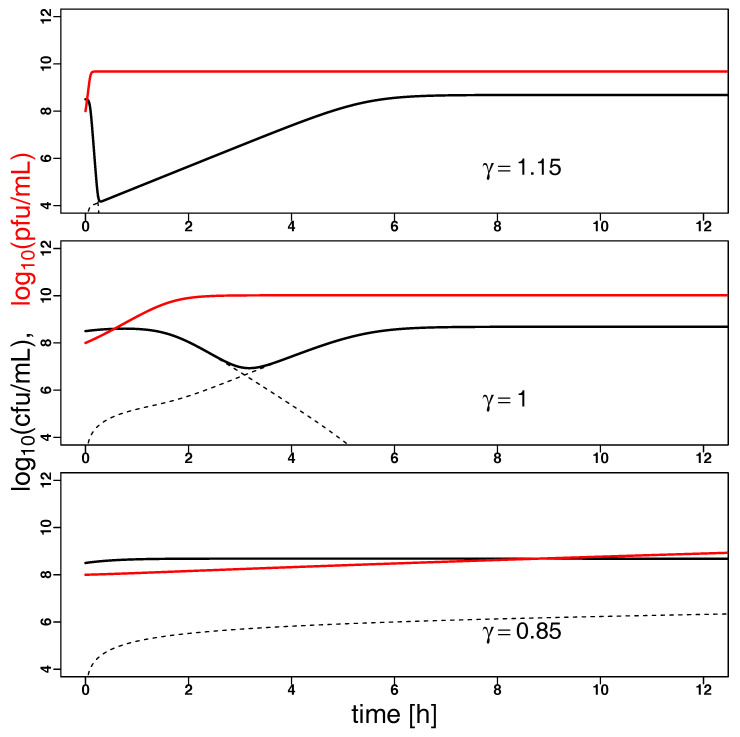
Phage synergy leads to fast bacterial depletion: The evolution of a bacterial species (solid black) and the corresponding phage (solid red) population sizes (in colony forming units (cfu mL${}^{-1}$) and plaque forming units (pfu mL${}^{-1}$)) over time (in hours) is extrapolated from the model based on the parameters *a*, *f*, *m*, *h*, and $K_{c}$ as estimated in Figure 2 and summarized in Table 1 with starting concentrations $10^{8.5}$ cfu mL${}^{-1}$ and $10^{8}$ pfu mL${}^{-1}$ and $b=5\times 10^{-10}$. Note that bacterial decline occurs later and less pronounced or is even impeded by prior emergence of resistant bacteria as γ (phage synergy) is decreased.

**Figure A2:**
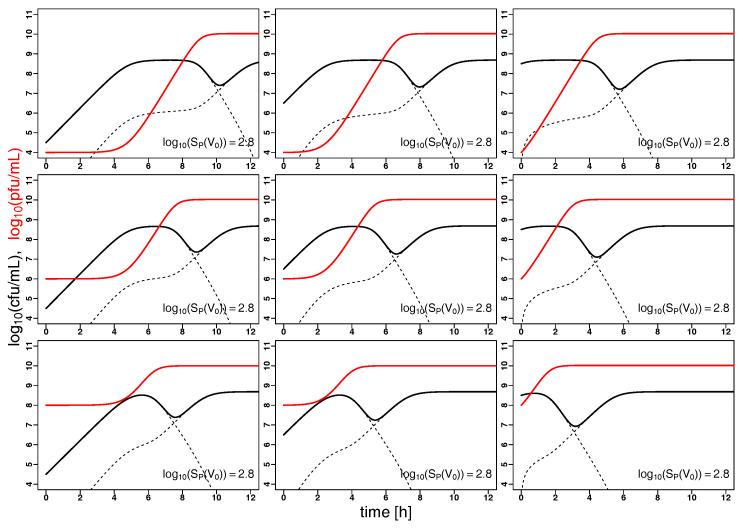
Population dynamics for bacteria and phages at γ=1: The evolution of *K. pneumoniae* (solid black) and the corresponding phage vB_KpnP_Lessing (solid red) population sizes (in colony forming units (cfu mL${}^{-1}$) and plaque forming units (pfu mL${}^{-1}$)) over time (in hours) is extrapolated from the model based on the parameters *a*, *f*, *m*, *h* and $K_{c}$ as estimated in Figure 2 with varying starting concentrations. As compared to Figure 3, phage binding has been reduced to mass action kinetics with γ=1 with a binding constant $b=5\times 10^{-10}$ corresponding to an effective binding $bV^{\gamma -1}$ in the above setting seen at $V=4.4\times 10^{5}\;{\mathrm{pfu}\;\mathrm{mL}}^{-1}=10^{5.6}$ pfu mL${}^{-1}$ (cf. middle row in Figure 3 for comparable initial phage concentration). Note that bacterial decline occurs later and less pronounced for mass action kinetics as compared to the synergistic kinetics with γ>1 shown in Figure 3.

Understanding the joint dynamics of *K. pneumoniae* and vB_KpnP_Lessing within the model based on the available experimental kinetic profiles (as shown in Figure 2) allows us to make predictions about the temporal evolution of bacterial and phage population sizes in other settings, e.g., at other initial concentrations of the given phage and its hosts or for characteristics of other hypothetical phage host pairs. Figure 3 shows such predictions for variable initial bacterial concentrations (per column) and initial phage concentrations (per row) with the right column corresponding to the setting in Figure 2, i.e., the characteristics of *K. pneumoniae* and its phage vB_KpnP_Lessing. Increasing the initial phage concentration from below the inundation threshold leads to a slower growth of the bacterial population reaching its minimum after depletion earlier. While bacterial load is reduced on average over time with growing initial phage concentration, the minimal bacterial load might increase near the inundation threshold for low initial bacterial concentrations. This is due to the accumulation of phage resistant bacteria in cases of strong phage-induced reduction of sensitive bacterial growth. Note the synergistic dynamics with γ>1 leads to a particularly fast decline of bacterial populations as compared to mass action kinetics represented by γ=1 (cf. Figure A1). Furthermore fixing γ=1 in the model led to kinetic fingerprint estimation which did not reflect the experimental data (cf. Figure A2 with corresponding parameters and γ=1). Synergistic dynamics (γ>1) leads to a decrease of the minimal phage concentration that is required to support bacterial depletion (inundation threshold $V_{I}$) and the minimal bacterial concentration needed for phage proliferation (proliferation threshold $S_{P}$) as can be seen in Figure 4 and in consequence supports bacterial depletion.

**Figure 5:**
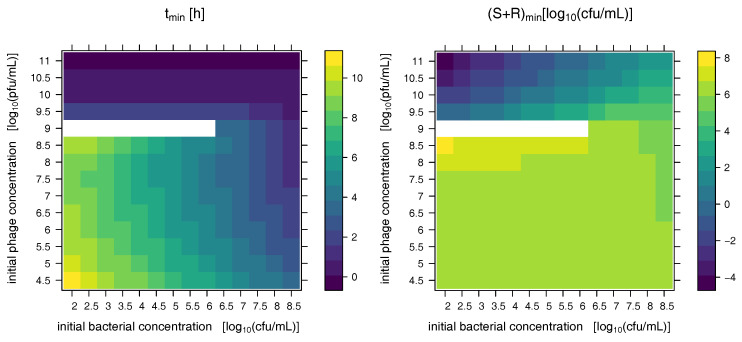
Combinations of initial bacterial and phage concentrations show multifaceted dynamics: The left panel shows the time (in hours) at which the bacterial concentration is minimal based on initial bacterial and initial phage concentrations. Model parameters are chosen as in Figure 2, whose initial conditions are reflected by the rightmost columns of each panel. Above the inundation threshold $V_{I}=10^{9.1}$ pfu mL${}^{-1}$ bacterial concentration declines immediately after phage addition resulting in a fast culmination of minimal bacterial load. If the initial phage dose is just below the inundation threshold sensitive bacteria are not depleted but only diminished in their growth which may result in no decline of bacterial load before resistant bacteria take over (white areas in the plots, starting concentrations represent minimal concentrations). This is also reflected in the minimal bacterial load observed before the rebound of resistant bacteria that is shown in the right column ($log_{10}$ of total bacterial concentration at its minimum).

These multifaceted dynamics in particular for low initial bacterial load treated with phages at concentrations just below the phage inundation threshold can further be studied in Figure 5 which shows the time at which a minimal bacterial concentration is observed as well as the minimal observed bacterial concentration after phage-induced bacterial depletion. Above the inundation threshold $V_{I}=10^{9.1}$ pfu mL${}^{-1}$ bacterial concentration declines immediately after phage addition resulting in a fast culmination of minimal bacterial load. If the initial phage dose is just below the inundation threshold sensitive bacteria are not depleted but only diminished in their growth which may result in no minimum of bacterial load before resistant bacteria take over (white areas in the plots). In consequence counter-intuitive behavior may arise for phage dosing schemes that approach the inundation threshold from below: The phage population grows slowly on small bacterial populations whose growth rate is increasingly diminished with growing phage populations while at the same time the population of resistant bacteria is rising. This way, the impact of phages on bacteria may be reduced with growing initial phage concentrations to be resumed only above the inundation threshold. At the inundation threshold, this may result in (essentially) no decline of bacterial load before resistant bacteria take over (white areas in the plots of Figure 5, starting concentrations represent minimal concentrations).

Especially for low initial bacterial concentrations high concentrations of phages are required to induce a noticeable bacterial depletion before resistant bacteria take over. Note that bacterial concentrations below one cfu mL${}^{-1}$ (negative values on logarithmic scale in Figure 5) can be expected to have a high probability of extinction due to stochastic effects.

**Figure 6:**
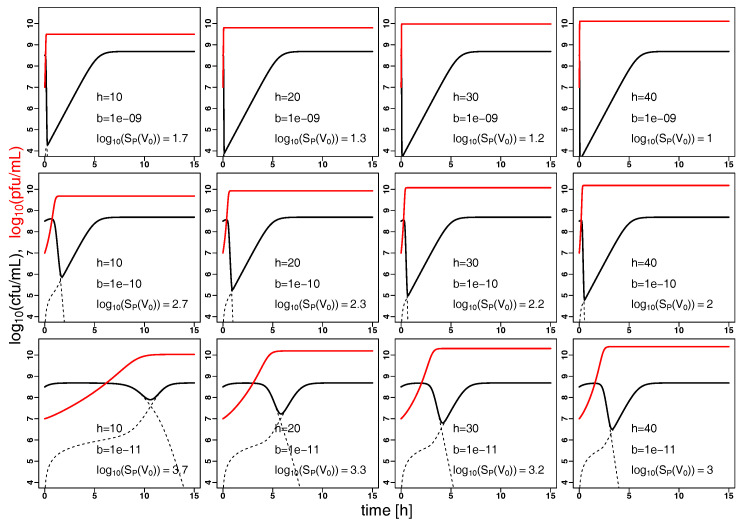
Phage binding has a strong impact on bacterial depletion: The evolution of bacteria (solid black) and the corresponding phage (solid red) population sizes (in colony forming units (cfu mL${}^{-1}$) and plaque forming units (pfu mL${}^{-1}$)) over time (in hours) is extrapolated from the model based on the parameters *a*, *f*, *m* and $K_{c}$ estimated in Figure 2 with starting concentrations $S_{0}=10^{8.5}$ cfu mL${}^{-1}$ and $V_{0}=10^{7}$ pfu mL${}^{-1}$. The binding parameter *b* is varied from $10^{-9}$ to $10^{-11}$ (by a factor of 1/10) from row 1 to 3, the effective burst size *h* is varied from 10 to 40 (in steps of 10) from columns 1 to 4. Dashed black lines correspond to the population sizes of phage sensitive and resistant bacteria. The initial concentrations of sensitive bacteria always exceed the proliferation threshold $S_{P}$ (cf. Equation (5)), i.e., the phage populations are growing. In the top row the initial phage concentration is above the inundation threshold $V_{I}$ leading to a rapid depletion of the bacterial population, in the middle row the inundation threshold $V_{I}$ is quickly reached whereas it takes longer in the bottom row for the phage population to reach the inundation threshold due to weak binding.

Furthermore, the model can extrapolate the dynamics of bacterial and phage populations for a hypothetical phage species that is characterized by different parameters. For an experiment comparable to that shown in Figure 2 (at MOI 0.1), we study in Figure 6 the variation of binding parameter *b* and phage burst size *h* acknowledging that the burst size is biologically more constrained (to a linear scale). In consequence, the variation of binding parameter *b* appears to influence bacterial burden strongly in comparison to the burst size *h* (Figure 6).

## 3. Discussion and Conclusions

Tailored phage therapy requires an understanding of the temporal evolution of the bacterial target population and the applied phage population which we refer to as kinetic fingerprint. Here we have studied such fingerprint of a human urinary tract *K. pneumoniae* isolate and the corresponding phage vB_KpnP_Lessing as modeling parameters have been estimated based on experimental data of the temporal evolution of bacteria and phage population sizes under culture conditions (Table 1 and Figure 1 and Figure 2).

The key finding from kinetic fingerprinting is that vB_KpnP_Lessing phage infection dynamics appears to be synergistic (as represented by γ>1). A fast and strong depletion of the bacterial host through synergistic interactions is a favorable feature for phage therapeutic approaches. More refined experiments and mathematical models will help to identify underlying mechanisms in more detail and to evaluate their potential. One mechanism may relate to complex stoichiometries [26] as some phage species can enter a bacterium at multiple sites whereas others are restricted to a single or few entry points. Another synergistic effect may arise through the activation of prophages, which are prevalent in most *Klebsiella* species [27]. In fact, Klebsiella is one of the most prophage-rich species, comparable to *E.coli* and *Yersinia enterocolitica* with a medium of nine prophages per genome [27]. The phages used in the current study belong to so called K32 phages [21,28]. A known characteristic of phage K32 and related phages which belong to *Podoviridae* is the presence of capsule-polysaccharide specific depolymerase. This enzyme for capsule degradation is required for adsorption to the host cell and subsequent degradation of the capsule in order to expose outer membrane components for phage binding [21,29,30]. The distant action of depolymerase on neighboring bacteria may contribute to the observed synergy. While we study synergy in a system with one phage species, we also realize that different phage species have been observed, showing that different species can deplete bacteria better in combination than alone [31]. Collective evasion of bacterial CRISPR immunity is another example of the synergistic interaction between phage species [19]. Finally, phage communication through quorum sensing and small-molecule communication can further add to the complexity [11], in particular relating to prophage induction [11,32,33].

Using the modeling of the kinetic fingerprint to extrapolate the observed dynamics to different regimes of phage dosing and bacterial load (Figure 3) shows the requirement of high phage concentrations, in particular, to control small bacterial concentrations (Figure 5). This is well known from the usage of bacteriophages in the food industry, such as for depletion of contaminations by pathogenic *Listeria monocytogenes* [34]. This finding is of particular importance in the light of the recent “PhagoBurn” clinical trial, in which the requirement for production of high concentrations of the therapeutic bacteriophages was reported [35]. To achieve effective bacterial depletion, phages have to bind and infect bacteria sufficiently well and produce enough offspring during bacterial lysis. These qualitative predictions are in line with intuition. As the model can also make quantitative predictions it can give advise on phage characteristics to meet pre-defined target values in terms of bacterial depletion. Some phage properties are natural characteristics and intrinsic to the phage strain, whilst others could be artificially modified and optimized. Our model suggests that phage binding has a major impact on bacterial depletion (Figure 6) which makes binding properties an interesting target for engineering of therapeutic phages [36].

Ecologic dynamics further differ between cases in which resistance arises from minority populations, from mutations or in which resistance is dominated by anti-phage immunity [37,38] and its evasion through resistance and phage synergy [19]. In consequence, more complex models may be required for some combinations of bacterial and phage species to describe the emergence of resistance—in turn informing about underlying mechanisms of resistance in a similar fashion as informing about phage synergy in the current study.

Additional environmental interactions such as anti-bacterial immunity in bacterial hosts [17,18] or anti-microbial interventions [25,39,40] may further influence the kinetics of phages and their bacterial host. This shows the importance to study bacteria phage systems in well defined settings as exemplified in our study to distinguish the influence of many potential contributions to the kinetic profile of more complex bacteria phage mixtures and their ecology. This simple mathematical framework can be used for classification of basic interactions between pairs of bacterial and phage species and from this naturally be extended to more complex systems involving more bacterial and phage species. The kinetic fingerprint introduced here provides a basis to describe more complex bacteria and phage communities and their dynamics as emergent from elementary interactions. This paves the way from descriptive to predictive phage ecology which we have shown to be highly relevant for a successful implementation of phage therapy.

## 4. Materials and Methods

### 4.1. Model Equations

We follow the arguments by Cairns et al. [14] and analyze the dynamics of populations of bacteria susceptible or sensitive to phage infection (*S*), those resistant to phage infection (*R*) and the population of free phage viral particles (*V*). Given compatibility with our data and the aim of a parsimonious model, we assume that phage resistance has only a minor impact on bacterial growth and introduce a joint growth rate *a* for sensitive and resistant bacteria that is however limited by the carrying capacity $K_{c}$ of the system. Mutations from sensitive to resistant bacteria occur at a rate *f*. In a similar fashion, we noticed that the latent period between infection of bacteria and cell lysis can be neglected within our model (i.e., K=0 in [14], data not shown). Phage binding has been modeled by the principle of mass action leading to degradation of sensitive bacteria (into infected bacteria) at a rate bV with a rate parameter *b* that can be associated with phage binding. However, earlier studies suggest that there is a concentration dependence of *b*, typically resulting in a reduction of the estimated parameter *b* with increasing phage concentration [14]. Other modeling approaches [17] have considered this explicitly as saturation effect or a side effect of heterogeneous mixing resulting in a rate $bV^{\gamma }$ with γ<1. Here, we look at degradation of bacteria at a rate $bV^{\gamma }$ only requiring γ>0. Phages have an effective burst size of *h* indicating the average number of productive phages released under bacterial lysis and are degraded at a rate *m*. The model is summarized in the following set of differential equations:

(1) $$\begin{array}{ccc} \frac{dS}{dt} & = & a\left(1-\frac{S+R}{K_{c}}\right)S-fS-bSV^{\gamma } \end{array}$$

(2) $$\begin{array}{ccc} \frac{dR}{dt} & = & a\left(1-\frac{S+R}{K_{c}}\right)R+fS \end{array}$$

(3) $$\begin{array}{ccc} \frac{dV}{dt} & = & \left(h-1\right)bSV^{\gamma }-mV. \end{array}$$

The concentration of sensitive bacteria can only decline if $\frac{dS}{dt}<0$ resulting in an inundation threshold, i.e., a minimal phage concentration that is required for the bacterial population to decline (cf. Figure 4).

(4) $$\begin{array}{ccc} V_{I}\left(S+R\right) & = & {\left(\frac{a\left(1-\frac{S+R}{K_{c}}\right)-f}{b}\right)}^{\frac{1}{\gamma }}<{\left(\frac{a-f}{b}\right)}^{\frac{1}{\gamma }}=V_{I}^{max}\\ V_{I}\left(S+R\right) & \approx & V_{I}^{max}\;\mathrm{for}\;S+R\ll K_{c}. \end{array}$$

On the other hand a minimal bacterial population is required for the phage population to grow, i.e., to have $\frac{dV}{dt}>0$, which is (cf. Figure 4)

(5) $$\begin{array}{c} S_{P}\left(V\right)=\frac{m}{b\left(h-1\right)V^{\gamma -1}}<\frac{m}{b(h-1)}=S_{P}^{max},\;\mathrm{for}\;V>1. \end{array}$$

Note that the proliferation threshold is independent of the size of the phage population in the case of mass action kinetics (γ=1). In case of γ<1 the proliferation threshold grows with the number of phages suggesting competitive dynamics or heterogeneous mixing. In case of γ>1 the proliferation threshold decreases with the number of phages suggesting synergistic dynamics.

### 4.2. Parameter Estimation

Numerical calculations were done using R statistical software (Version 3.6.2) using the package deSolve (Version 1.27.1) for numerically solving the differential Equations (1)–(3). Optimal parameters were estimated by minimizing the sum of quadratic differences between experimentally observed values and those predicted by the differential Equations (1)–(3) on logarithmic scale for both bacteria and phage populations. Optimization was done by using the ‘L-BFGS-B’ algorithm implemented in the function optim from the R stats package. Parameter estimates were done independently for 50 initial conditions drawn from uniform distributions within predefined ranges (for *f*, *b*, *m*, $K_{c}$ on logarithmic scale). As averaged or median values of these estimated parameters shown in Table 1 are not generally optimal themselves we rather refer to the parameter set with the lowest sum of quadratic differences attained in our optimisation procedure as the estimated optimal parameter set. R code is available upon request.

### 4.3. Experimental Methods

#### 4.3.1. Bacteria and Phage Strains

*Klebsiella pneumoniae* strain DSM11678 was originally isolated from human urinary tract and obtained from the German Collection of Microorganisms and Cell Cultures (DSMZ). The corresponding *Klebsiella* phage vB_KpnP_Lessing (DSM107143) was isolated from sewage and also provided by DSMZ. Sequencing of vB_KpnP_Lessing (Johannes Wittmann, unpublished) and BlastN analysis revealed that the most related genome sequence in the database was Klebsiella phage K5-2 (accession KY389315.1, 92% query coverage and 95% identity [41]). vB_KpnP_Lessing produces 2–3 mm diameter clear plaques surrounded by halos.

#### 4.3.2. Growth and Enumeration of Bacterial Cells and Infectious Phage

*Klebsiella pneumoniae* strain DSM11678 was grown in 50 mL liquid Nutrient Broth Medium (Sigma-Aldrich) supplemented with 5 mM ${\mathrm{CaCl}}_{2}$ and 5 mM ${\mathrm{MgSO}}_{4}$ at 37 °C with 120 rpm agitation using 100 mL conical flasks, or on solid medium plates supplemented with 1.5% agar (Sigma-Aldrich). For phage propagation bacteria were grown to log phase, approximately $10^{8.5}$ cfu mL${}^{-1}$, at which time point phage was added to an approximate MOI of 1 or below as indicated. Incubation with shaking was continued and samples of the culture removed at various time points for measurements. Bacteria were enumerated using the SphereFlash spiral plater (IUL). For enumeration of phages bacterial cells were pelleted by centrifugation at 6000× *g* and further removed by filtration using a 0.22 μm filter. Phage suspensions were titrated using the double agar layer technique. For this approximately 10^8^ cfu mL^−1^ of a log-phase growing bacterial culture and serial dilutions of phage suspensions were added to prewarmed topagar (0.5% agar), and mixed and poured on medium plates. Plaques were counted after overnight incubation at 37 °C.

## Figures and Tables

**Table 1:** Optimal model parameters: Optimal model parameters were estimated for the data shown in Figure 1 from 50 sets of random starting values (cf. Materials and Methods), followed by mean estimated model parameter values (with standard deviation) among 10 most highly ranked and all model parameter estimates. The binding exponent γ=1.15>1 indicates synergistic phage dynamics.

	*a*	*h*	γ	$\log_{10}\left(f\right)$	$\log_{10}\left(b\right)$	$\log_{10}\left(m\right)$	$\log_{10}\left(K_{c}\right)$
optimal	2.0	15	1.15	−3.4	−10	−5.3	8.7
top 10	2.0±0.2	15±0.6	1.15±0.01	−3.5±0.2	−10±0.1	−5.9±1.4	8.7±0.03
all	1.7±0.7	13±2	1.12±0.06	−3.2±0.5	−9.8±0.5	−6.2±1.6	8.7±0.12

## Abbreviations

The following abbreviations are used in this manuscript:

cfu	colony forming units
pfu	plaque forming units
moi	multiplicity of infection
MDR	multi drug resistant/resistance
